# *Chlamydophila psittaci* Transmission from Pet Birds to Humans

**DOI:** 10.3201/eid1307.070074

**Published:** 2007-07

**Authors:** Daisy Vanrompay, Taher Harkinezhad, Marijke van de Walle, Delphine Beeckman, Caroline van Droogenbroeck, Kristel Verminnen, Ruud Leten, An Martel, Katty Cauwerts

**Affiliations:** * Ghent University, Ghent, Belgium; † Ghent University, Merelbeke, Belgium; ‡ Federal Department of Health, Food Chain Safety and Environment, Brussels, Belgium

**Keywords:** Chlamydophila psittaci, psittacosis, zoonosis, pet bird, genotyping, PCR, Psittaciformes, dispatch

## Abstract

We studied zoonotic transmission of *Chlamydophila psittaci* in 39 breeding facilities for Psittaciformes (cockatoos, parrots, parakeets, lories) that frequently used antimicrobial drugs. Genotypes A or E/B were detected in 14.9% of humans at these facilities. Information on antimicrobial drug use in Psittaciformes and a *C. psittaci* vaccine are urgently required.

*Chlamydophila psittaci*, an obligate, intracellular, gram-negative bacterium, has 7 known genotypes (A–F and E/B) (*1*), All genotypes can be transmitted to humans and cause psittacosis or parrot fever (*2*). Genotypes are distinguished by sequencing of the outer membrane protein A (*ompA*) gene (*3*) or by a recently developed *ompA* genotype-specific real-time PCR (*4*). *C*. *psittaci* can infect 465 avian species in 30 avian orders, with at least 153 species in the order Psittaciformes (*5*). From 1988 through 2003, a total of 935 human cases of psittacosis were reported to the US Centers for Disease Control and Prevention (*6*); most were related to contact with Psittaciformes. Currently, ≈100 psittacosis cases are reported annually in the United States, and 1 person may die of this disease each year. The incidence of psittacosis in men seems to be increasing in industrialized countries and is related to importation of exotic birds. Other cases may not be correctly diagnosed or reported.

## The Study

We investigated zoonotic transmission of *C*. *psittaci* in Belgian breeding facilities for Psittaciformes (cockatoos, parrots, parakeets, lories). Participants were recruited through the Belgian Society for Parakeet and Parrot Breeders. Fifty breeding facilities received a sampling package by regular mail. The package contained a questionnaire designed to assess information on the pet bird owners’ professional and nonprofessional activities, smoking habits, general health status, use of medication, allergies, clinical signs specifically related to psittacosis; on the birds’ origin, housing, feeding, breeding, health status, and medication; and on the presence of other bird species. The package also contained dacron-tipped swabs and instructions for fecal (cage floor) or pharyngeal sampling in birds or humans, respectively. We provided transport medium (*7*) for isolation and DNA stabilization buffer (Roche, Brussels, Belgium) for PCR, to be added to swabs after sampling.

Packages returned by express mail 1 day after sampling were stored at –80°C until use. Sampling packages are convenient and safe for investigators. Forty-one (82%) of 50 breeding facilities returned the packages. Two packages were incomplete and therefore excluded, resulting in samples from 308 birds and 46 humans from 39 Psittaciforme breeding facilities for testing. We also obtained pharyngeal samples from a veterinary student who was involved in another study at the same breeding facilities. This student was examined before our study and every month for 4 months. All humans were examined and provided informed consent.

The *ompA* gene was detected in birds and humans by using a *C*. *psittaci*–specific nested PCR/enzyme immunoassay (EIA) (*8*). Viable *C*. *psittaci* were detected by isolation in buffalo green monkey cells and direct immunofluorescence staining (IMAGEN; Dakocytomation, Copenhagen, Denmark) (*7*). When zoonotic transmission occurred, the infection source was traced by using a genotype-specific real-time PCR (*4*) for specimens from birds and their owners.

Fifty-nine (19.2%) of 308 Psittaciformes were positive for *C*. *psittaci* in the nested PCR/EIA, and bacteria were isolated from 25 (42.3%) birds with PCR-positive results. Of 39 tested breeding facilities, 8 (20.5%) were positive in both the nested PCR/EIA and culture, and respiratory disease was present at all facilities. Five other breeding facilities showed only positive results for the nested PCR/EIA. One of these facilities was currently treating birds with doxycycline, and the remaining 4 had recently used doxycycline, oxytetracycline, or enrofloxacin. Treatment was successful because viable *C*. *psittaci* were not detected at these 5 breeding facilities, and all their birds appeared healthy. A total of 13 (33.3%) of 39 *C*. *psittaci*–positive breeding facilities showed a significant correlation between fecal excretion of viable chlamydia and respiratory disease (odds ratio 14.5, 95% confidence interval 1.6–130.5, p<0.05).

The remaining 26 breeding facilities with healthy birds had negative results for PCR and culture. Early infections may have been missed because fecal excretion occurs after primary bacterial replication in the respiratory tract and septicemia. Moreover, fecal shedding occurs intermittently and healthy carrier birds might not excrete bacteria for >1 year. Pharyngeal swabs would have been a better choice for testing but psittacine owners are reluctant to catch their birds because stress induces respiratory disease. Reactivation of a *C*. *psittaci* carrier status might be involved in this phenomenon.

Nested PCR/EIA detected *C*. *psittaci* DNA in 6 (13%) of 46 pet bird owners. All 6 owners obtained birds at different breeding facilities, and all facilities had birds positive for *C*. *psittaci* by PCR and culture. The infected persons were 22, 24, 31, 38, 49, and 56 years of age (mean age of the pet bird owners was 46 years). Viable organisms were present in 4 of 6 persons with positive PCR results, and all 4 (who were nonsmokers and had no allergies) had mild respiratory illness (shortness of breath or rhinitis and coughing). Although the study was conducted in the summer, 8 (20%) of the remaining 40 *C*. *psittaci*–negative owners reported rhinitis or coughing during the past 2 weeks, some (7.5%) with a sore throat. Thus, statistical conclusions on the relationship of viable chlamydophila in humans and respiratory disease were not obtained.

The 23-year-old veterinary student was negative for *C*. *psittaci* before the study. His first pharyngeal specimen after he visited breeding facilities was positive by both PCR and culture. He remained infected until 1 month after ending his study but showed only mild respiratory illness.

*C*. *psittaci* genotype A was found in 5 pet bird owners and the veterinary student; these persons had rhinitis and a cough. The strain isolated from the pet bird owner who reported continued shortness of breath was genotype E/B. Thus, in contrast to the recently published reports on human psittacosis and pet birds (*9*–*11*), severe clinical signs with pneumonia did not occur in these patients and none were treated. However, 10 (25.6%) of 39 bird owners mentioned in the questionnaire that they had pneumonia after keeping Psittaciformes as pets, which was higher than the yearly rate of 8/1,000 pneumonia cases in Belgium. It is likely that pet bird owners and veterinarians are regularly infected and protected against severe disease. However, whether they become carriers and the possible consequences of infection are unknown. Protective clothing, including air filter face masks, is recommended for preventing occupational disease.

## Conclusions

In our study, 18 (46.2%) of 39 breeding facilities had treated their birds with tetracycline, doxycycline, or enrofloxacin in the past year. Four (10.2%) of 39 also used tetracyclines prophylactically. Of these 18 facilities, 8 (44%) were positive for *C*. *psittaci* by nested PCR/EIA and 3 (16.6%) were positive by culture. Because of the risk of developing drug-resistant strains, as described for *Chlamydia suis* (*12**,**13*), regular use of antimicrobial drugs must be avoided. Since there is no vaccine against psittacosis, pet bird owners frequently use tetracyclines for treatment or prevention of respiratory disease. These drugs are sold on the Internet without a prescription because a prescription is not needed in every country. Before the advent of antimicrobial drug treatment, human mortality rates were 15%–20%. Thus, a vaccine and information on sensible use of antimicrobial drugs in Psittaciformes are needed to prevent psittacosis in humans and development of drug-resistant bacterial strains.
